# Psychiatric Comorbidities in Children With ASD: Autism Centre Experience

**DOI:** 10.3389/fpsyt.2021.673169

**Published:** 2021-06-09

**Authors:** Iva Ivanović

**Affiliations:** Clinic for Psychiatry, Clinical Centre of Montenegro, Podgorica, Montenegro

**Keywords:** autism spectrum disorder, psychiatric comorbidity, epidemiology, neurodevelopmental disorders, cross-sectional study

## Abstract

Autism spectrum disorder (ASD) is a neurodevelopmental disorder with social communication deficits, restricted interests, and repetitive behaviours. In this lifelong condition the core features that cause impairment may also be expanded by behavioural and emotional problems. Individuals with ASD are likely to experience a higher prevalence of common mental disorders compared to the typically developed individuals. This high epidemiological burden of various psychiatric disorders among ASD population encourages further research and improvement in diagnostic practise in ASD and comorbid disorders. In this brief research report of a cross-sectional study, I aimed to estimate the psychiatric comorbidity prevalence and describe their general characteristics in children with ASD in the Autism Centre in Montenegro. The study population consisted of 152 patients who were diagnosed with ASD, 117 male and 35 female, and the mean age (SD) was 8.02 (4.26). In this brief research report prevalence of children with ASD with at least one psychiatric comorbidity was 36.84%. Only one psychiatric comorbidity disorder was reported in 17.16%, two in 9.87%, three in 8.55%, and in 0.66% patients four other psychiatric disorders. Psychiatric disorders present in this population sample were attention deficit hyperactivity disorder (17.76%), conduct disorder (13.10%), disruptive mood dysregulation disorder (9.87%), anxiety disorder and insomnia (7.89%), elimination disorder (3.29%), and depression (1.97%).

## Introduction

Autism spectrum disorder (ASD) is a neurodevelopmental disorder characterised by social communication deficits, restricted interests, and repetitive behaviours (1). In 2012 a review of global prevalence estimates of autism spectrum disorders found a median of 62 cases per 10,000 people (2).

As an early-onset disorder, with a chronic course ASD represents a lifelong condition which affects lives not only of the affected individuals but also of their families, caregivers, and communities (3). In this lifelong course the core features of ASD that cause impairment may also be expanded by behavioural and emotional problems (4, 5). Past research showed that ASD can co-occur with other psychiatric disorders (6, 7). Additionally, the issue of psychiatric comorbidity in ASD has become more relevant in recent years as the *Diagnostic and Statistical Manual of Mental Disorders* (*DSM-5*) no longer excludes additional diagnoses among individuals with ASD (8).

Individuals with ASD are likely to experience a higher prevalence of common mental disorders compared to the typically developed individuals (8, 9), and it is estimated that nearly 70% of people with ASD experience at least one comorbid psychiatric disorder, whereas nearly 40% of individuals may have two or more psychiatric disorders (9). With increasing prevalence of various mental disorders worldwide, a comprehensive evaluation of the prevalence of co-occurring psychiatric disorders among individuals with autism spectrum disorder occurred through various reviews, meta-analysis and umbrella reviews. The latest umbrella review showed variation of the prevalence of comorbid disorders among people with ASD across reviews, from 54.8% (95% CI: 46.6–62.7) to 94% (10–12). Reasons for this variation in prevalence of studies could be found in comparing population subgroups as well as for different methodologies and instruments used to assess those disorders. Comparing age-population subgroups individuals above 18 years had a higher prevalence of psychiatric comorbidity compared to younger participants (13). Also, children with ASD have been found to have a higher burden of psychiatric comorbidity than children with intellectual disability (8). Moreover, studies with smaller sample sizes and instruments like *DSM-III* or later and ICD-10 had higher prevalence compared to studies with larger samples and older instruments (13).

The prevalence of co-occurring attention deficit/hyperactivity disorder (ADHD) among people with autism ranged from 25.7 to 65% (11, 14, 15), while disruptive, impulse-control, and conduct disorders ranged from 12 to 48% (12, 15, 16), the prevalence of obsessive-compulsive disorders (OCD) co-occurring in ASD, which ranged from 9 to 22% (8, 17, 18), the prevalence of suicidal ideation and attempts, which ranged from 10.9 to 66% and 1 to 35%, respectively (12, 14, 16, 19, 20). Sleep-wake disorder was 13% (95% CI: 9–17) among 190,963 participants with ASD (15), schizophrenia spectrum, and other psychotic disorders was reported in range from 4 to 67% (11, 13, 15, 16, 21–23). Five reviews reported the prevalence of mood disorders, which ranged from 4.4 to 37% across ASD samples (11, 12, 16, 22, 24). Eight reviews reported the prevalence of anxiety disorders among people with ASD, which ranged between 1.47 and 54% across studies (11, 12, 15, 17, 18, 22, 25). The prevalence of depressive disorders among individuals with ASD was ranged from 2.5 to 47.1% (15, 17, 24, 26–28).

The aim of this brief research report of cross-sectional study is to estimate the psychiatric comorbidity prevalence and describe their general characteristics in children with ASD in the Autism Centre in Montenegro. To my best knowledge this is the first study about ASD children population and comorbid psychiatric disorders in Montenegro.

## Methods

This is a cross-sectional study of present psychiatric comorbidity in a children with autism spectrum disorder. The study population derived from medical records present in the health-care institution Autism Centre in Podgorica, Montenegro. The inclusion criteria were all patients who were diagnosed with autism spectrum disorder (ASD). These diagnoses had been coded according to ICD-10. Three autism spectrum diagnoses were found: autistic disorder, atypical autism, and Asperger syndrome. ASD is used here for all three categories. Diagnosis of ASD was defined by both clinical and instrument diagnostic assessment, Autism Diagnostic Interview–Revised (ADI-R) and Autism Diagnostic Observation Schedule (ADOS), that was confirmed in their medical records. Since there is no official register for all patients of the Centre, revising medical records and searching for eventual other comorbidities was needed. Detailed revision has been done, and all psychiatric disorders other than ASD in these patients were marked. Diagnoses for these psychiatric disorders were done mostly using clinical examination, interviewing parents, and in suitable occasions using teachers and school reports (or care-centre). Instead of using ICD-10 coding for hyperkinetic disorder, we used more commonly used attention-deficit-hyperactivity disorder (ADHD), as described in medical records. Also, *DSM-V* diagnosis for disruptive mood dysregulation disorder (DMDD), described as severe ongoing irritability, anger, and frequent, intense temper outbursts, was a better fit for diagnosis of equally the same described disorder in some patients than ICD-10's mixed emotions and behaviour disorder. Elimination disorder was used for enuresis and encopresis together. Characteristics of the patients, their parents, pre/peri/post-natal period, instrumental exams, and pharmacological treatment, if available in medical records, were assessed. Data for gender, age, number of siblings and twins, maternal and paternal age at moment of child's birth, assisted reproductive technology, positive psychiatric heredity, and mother's illness before and during pregnancy, pregnancy complications, labour and delivery complications, caesarean section, gestation age, Apgar score, and early postnatal complications were assessed for every patient of this study sample. Birth weight data wasn't available for every patient. Although electroencephalogram (EEG) was not performed for every patient, for those ASD patients with other psychiatric disorder, their EEG exam result was reported, as well as potential epilepsy diagnosis, if they were available in medical records. The same goes for magnetic resonance imaging (MRI); if available, results for ASD patients with other psychiatric disorders were mentioned.

Data analysis was performed by EZR (Easy R) software. Descriptive statistics were calculated for all variables of interest and included means and standard deviations for continuous measures or counts and percentages for categorical data. Fisher's test was used for odds ratio for the confidence interval of 95%.

The study was approved by the Ethical Committee of Clinical Centre of Montenegro (03/01-21682/1).

## Results

### General Description of a Study Population

This study population consisted of 152 patients who were diagnosed with ASD, 117 male and 35 female. The mean age (SD) was 8.02 (4.26) (Table 1). Twelve are siblings and four pairs of these siblings are twins. Parents of these patients, mothers and fathers, at the moment of their children's birth, had mean age (SD), respectively of 30.60 (5.40) and 35.19 (8.09), with percentage of age range (Table 2). In the medical records positive psychiatric heredity was notified in 10 patients. Assisted reproductive technology helped five patients to be conceived. Regarding prenatal circumstances, during their pregnancies 14 mothers reported having chronic medical morbidities, while for the reasons of the acute or chronical medical disorders 13 mothers were treated with pharmacological therapy. Sixty patients were delivered by caesarean section. Delivery and labour related complications had happened in 22 patients and they were mostly described by perinatal asphyxia, haemoraggia, and the birth trauma caused by the assistance of vacuum. Eight patients suffered postnatal complication (clavicular fracture and hypertonia). Preterm birth happened in 19 of our patients, but almost all of them were born in the late preterm period. Low Apgar score was notified in eight patients.

**Table 1:** Presents general study data.

**Characteristics**	**ASD**	**ASD with comorbidity**
Total number	152	56 (36.84%)
Male	117 (76.97%)	40 (71.42%)
Female	35 (23.03%)	16 (28.57%)
Mean age	8.019 sd 4.26	10.48 sd 4,30
Siblings	12	One of the siblings
Twins	8	0
IQ > 70	21 (13.81%)	10 (17.86%)
Assisted reproductive technology	5 (3.39%)	2 (3.57%)
Psychiatric heredity	10 (6.58%)	2 (3.57%)
Preterm born	19 (12.50%)	8 (14.29%)
Low birth weight <2,500g	10 (6.57%)	3 (5.36%)
Caesarean section	60 (39.47%)	25 (41.67%)
Mother's medical disorder	14(9.21%)	6 (10.71%)
Mothers taking medicines	13 (8.55%)	5 (8.93%)
Pregnancy complications	6 (3.95%)	3 (5.36%)
Delivery complications	22 (14.47%)	12 (21.43%)
Apgar score <7	8 (5.26%)	2 (3.3%)
Postnatal complications	8(5.26%)	3 (5.36%)

**Table 2:** Presents age of parents.

**Parental age**	**ASD *n* (% of tot ASD)**	**Mean age (SD) in only ASD**	**Comorbidities n (% of ASD comorbidity sample)**	**Mean age (SD) in ASD comorbidity sample**
Mother's		30.60% (5.40)		30.25%(4.48)
>30	66 (43.42%)		25 (44.64%)	
30–35	57 (37.5%)		25(44.64%)	
36–40	22 (14.47%)		6 (10.71)	
<40	7 (4.61%)		0	
Father's		35.19 (8.09)		35.82 (4.48)
>30	25 (16.45%)		8 (14.28%)	
30–35	70 (46.05%)		25 (44.64%)	
36–40	25 (16.45%)		8 (14.28)	
<40	31 (20.39%)		15 (25%)	

### Psychiatric Comorbidities in ASD Patients

This brief research report of a cross-sectional study estimates the presence of at least one comorbid psychiatric disorder in 36.84% of children with ASD. Only one comorbid psychiatric disorder was reported in 17.16%, two in 9.87%, three in 8.55%, and four in 0.66% patients. Psychiatric disorders present in this population sample are ADHD reported in 17.76% of patients, conduct disorder (13.10%), disruptive mood dysregulation disorder (9.87%), anxiety disorder and insomnia (7.89%), elimination disorder (3.29%), and depression (1.97%) (Table 3).

**Table 3:** Presents presence of comorbidity disorders in study population.

**Comorbidity disorders**	***N* (%)**
ADHD	27 (17.76%)
Aggressive (conduct) disorder	19 (13.10%)
Disruptive mood dysregulation disorder	15 (9.98%)
Anxiety disorder	12 (7.89%)
Insomnia	12 (7.89%)
Elimination disorder	5 (3.29%)
Depression	3 (1.97%)
Enuresis/encopresis	5 (3.29%)

### General Description of ASD With Comorbidity Subsample

The mean age (SD) of the ASD patients who have psychiatric comorbidity is 10.3 (4.58). Patients with IQ lower than 70 in this ASD with comorbidity subsample are 18.33%. Maternal and paternal mean age (SD) were 30.25 (4.48) and 35.82 (4.48). Positive psychiatric heredity is reported in three patients. Two patients were conceived with the help of assisted reproductive technology. During pregnancies four mothers suffered from chronical medical disorders (hypertension, diabetes mellitus, asthma), two reported stressful life-situation, and five were under pharmacological treatment. Additionally, during the pregnancy course three mothers were diagnosed placenta previa and they needed correspondent treatment. Eight patients were preterm born, and in three of them low birth weight was reported. Perinatal complications like perinatal asphyxia, haemoraggia, and birth trauma caused by the assistance of vacuum were reported in 12 patients. Two patients had low Apgar score. In the early postnatal period three had problems with muscular hypertonia that required further treatment. Epilepsy was neurological comorbidity in eight patients with ASD and other psychiatric comorbidities. Although not every patient had an EEG exam, in those that had reported EEG assessment, another 17 patients had epileptiform discharges in absence of clinical seizure, while another six in their EEG result had changes but not enough specific for epileptiform activity. Also, MRI assessment was not indicated in every patient; in three patients with ASD and other psychiatric comorbidities, cortical and subcortical changes were found.

### Attention Deficit Hyperactivity Disorder and ASD

ADHD was found in 27 (17.76%) patients. This was the most frequent comorbidity in patients with ASD. The mean age (SD) was 9.58 (3.95). Gender distribution was 21 males and 6 females. One patient had IQ lower than 70. Since it was most frequent comorbid disorder in this study sample it was also estimated as concomitant psychiatric diagnoses in children with and without ADHD in our sample. It shows significant correlation with conduct disorder, anxiety, disruptive mood dysregulation disorder, and elimination disorder (Table 4). Five patients had epilepsy, in eight patients EEG exam detected epileptiform discharges in absence of clinical seizure, and five had changes but not enough specific for epileptiform activity. One patient had abnormalities on the right frontal lobe of the brain. Two patients were conceived with the assistance of reproductive technology and one patient had positive psychiatric heredity. Five mothers during pregnancy period had some chronic (diabetes mellitus, hypertension) or acute disorder (urinary infection) and had to use medical treatment. In three mothers placenta previa was diagnosed. Three patients were born in preterm period, one of them with low birth weight. Five patients had delivery complications (asphyxia) with consequent hypertonia in one patient. Assessing the ASD symptomatology using the ADI-R and ADOS instruments, four patients with ADHD had more pronounced impairment in social communication, comparing to the repetitive and stereotypic behaviour domain that was borderline in these patients. Predominantly hyperactive/impulsive presentation was described in 15 of 27 patients, 7 patients had predominantly inattentive presentation, and combined presentation was present in 5 patients. In medical records, very often there were described difficulties that these patients found in the school system, social interaction, but also in family environment. In a written description of their regular medical visits, these difficulties negatively impacted their life. Just a few patients had methylphenidate as their pharmacological therapy, while the others had predominantly second generation antipsychotic (risperidone and aripiprazole).

**Table 4:** Presents associations between ADHD and other diagnostic condition.

	**ADHD**	**No ADHD**	**Adjusted odds ratio**	
CD	6	13	2.44 (0.682–7.8949)	*P* = 0.1098
Anxiety	3	9	1.60 (0.2605–0.782)	*P* = 0.447
Insomnia	2	10	0.9204	*P* =1
DMDD	3	12	1.17(0.169–3.9617)	*P* =0.197
ED	2	3	3.21 (0,256–29,650)	*P* = 0.216
Depression	0	3	0 (0.00–11.388)	*P* = 1

### Conduct Disorder

Conduct disorder was reported in 19 (13.10%) patients. Mean age (SD) was 10.31 (3.97). Gender distribution was 14 males and 5 females. This disorder was mostly associated with ADHD (six patients), and four patients also had insomnia and three anxiety. Epilepsy was diagnosed in two, four had epileptiform discharges in absence of clinical seizures, and one had non-specific epileptiform activity. MRI described in one patient supratentorial lesions; this patient additionally has anxiety disorder, while in two others patients MRI detected changes in the parieto-occipital lobe.

Few pre/perinatal complications were described in their medical records, like mother's hypertension, diabetes, and placenta previa during pregnancies, perinatal asphyxia and birth trauma during labour, and muscular hypertonia as postnatal complication. Two of them were born in preterm period. In the ADIR and ADOS evaluation, three patients of this group had more pronounced social communication problems. Symptoms of aggression against others and destruction of items were described mostly in school and day-time centres, but also in family and familiar settings. Various pharmacological treatment was prescribed and many treatment strategies had been changed. Pharmacological treatment mostly comprehended typical and atypical antipsychotics and benzodiazepines.

### Disruptive Mood Dysregulation Disorders

Disruptive mood dysregulation disorder was present in 15 patients. Mean age (SD) was 11.29 (4.72). Gender distribution was 11 males and 4 females. Six of these patients had second psychiatric disorder other than ASD. In three patients DMDD was also associated with ADHD, while three patients had one of these disorders: anxiety disorder, conduct disorder, and elimination disorder. None of these patients had epilepsy but epileptiform discharges in absence of clinical seizures were found in three patients. No specific prenatal and perinatal circumstances were described.

### Insomnia

Insomnia disorder was present in 12 (7.89%) patients with ASD. Mean age (SD) was 10.72 (4.38). Gender distribution was eight males and four females. Patients with another psychiatric disorder were mostly the ones with conduct disorder, anxiety disorder, and ADHD, respectively, in four, three, and two patients. Pregnancy complications were found mostly in these patients. Mother's hypertension, diabetes mellitus, asthma, stressful life events, as well as the presence of placenta previa was followed by labour difficulties, as haemoraggia and hypoxia. Two patients were preterm babies. Epilepsy was diagnosed in one patient, while two patients had epileptiform activity without clinical seizure discharges. ADIR and ADOS evaluation showed that two patients had noticeable impairments in social domain. These patients mostly had melatonin as their medical treatment.

### Anxiety Disorder

Anxiety disorder was described in 12 (7.89%) patients. Mean age (SD) was 13.2 (3.73). Gender distribution was equal for male and females. The anxiety disorder in our sample was equally co-occurring with some other disorder (ADHD, conduct disorder, and disruptive mood dysregulation disorder). Two patients had epilepsy while three others had epileptiform activity without clinical seizure discharges. MRI showed abnormalities in the frontal right lobe of the brain in one patient. This patient also had associated ADHD and epilepsy. Pharmacological treatment was various and other comorbidities impacted these treatment variety. There were no available data on eventual psychotherapeutic treatment.

### Other Disorders

Elimination disorders (enuresis/encopresis) were reported in five patients. Mean age (SD) was 11.4 (4.63). Gender distribution was four male and one female (ration 4:1). No specific pre/perinatal complications were noted. Two patients also had ADHD disorder. Depression was diagnosed in three patients. Mean age was 12.3 (3.97). Gender distribution was three male and one female (ratio 3:1). No specific pre/perinatal complications were noted. Only one patient had on the EEG exam changes that were not specific enough for epileptiform activity. All patients with depression had pharmacological treatment that comprehends the use of serotonin selective reuptake inhibitors.

## Discussion

This brief research report is part of a cross-sectional study that is the first study of comorbid psychiatric disorders in children with ASD in Montenegro to date. Study population was described with all the available data of general characteristics, such as age and gender, as well as date of prenatal, perinatal, and postnatal period of the patients. If they were available, EEG and MRI exams, intellectual disability, and epilepsy were also brought to attention. In this brief research report prevalence of children with ASD with at least one psychiatric comorbidity was 36.84%. Those with one psychiatric disorder other than ASD were 17.16%, two 9.87%, three 8.55%, four 0.66%. These findings are below the prevalence found in the latest meta-analysis and reviews (54.8 to 94%) (10). To explain this difference a few facts need to be accentuated that at the same time represent the limits of this study. This is a cross-sectional study of a small sample population of only children. The majority of comorbid diagnoses were reported meeting the clinical criteria for diagnosis and not using standardised diagnostic instruments. Mean age (SD) of general study population was 8.019 (4.26) and in the comorbidity sample 10.3 (4.58). These findings were similar to those in literature reviews of children with ASD and comorbidities (29).

Autism spectrum disorders (ASD) and attention deficit hyperactivity disorder (ADHD) are the most studied neurodevelopmental disorders (30). Their symptoms very often overlap so we can find in both disorders difficulties in attention, communication with peers, impulsivity, and various degrees of restlessness or hyperactivity. In addition, both are more common in boys than in girls, and mostly present at pre-school age. Genetic pre-disposition is assumed for both disorders. In the last decades many studies have reported increased prevalence of ADHD and ASD (31–33), but also studies show that between 30 and 50% of individuals with ASD manifest ADHD symptoms (34). When *DSM-V* implemented changes in allowing the ADHD/ASD comorbidity, studies started to grow and some of the latest results talk about the comorbidity prevalence of 25.7 to 65% (11, 14, 15). Prevalence in this brief research report of children with both ASD and ADHD is 17.76%. In ADHD are also common comorbid psychiatric disorders like disruptive behaviour disorders (oppositional defiant disorder and conduct disorder) (35). This brief report also finds significant association in ADHD patients and conduct disorder. As literature suggests, epilepsy in ADHD patients is frequent (23–40%) (36). In our sample five patients had diagnosed epilepsy, while eight had epileptiform activity without clinical seizure charges. Abnormalities in fronto-striatal circuits have frequently been implicated in ADHD, and imaging studies have supported the presence of abnormalities in these circuits in persons with ADHD (37). In our sample we had one patient with abnormalities in the brain frontal lobe. But not every patient had done an MRI exam. Also, in this brief research report, some of the risk factors such as preterm birth and low birth weight were found, respectively, in three and one patient. But the data were not adjusted and not available for every patient. This will be one of the future research steps in this ongoing study.

In one systematic review conduct disorders were present in 12% of patients with ASD, which is similar to our findings where conduct disorder is present in 13.10% of patients (15). As mentioned before, this brief report found that 6 of 19 patients with conduct disorder also had ADHD. It is assumed that these patients had worse impact on quality of life, regarding social relationships. The main domains of ASD are impairment in socialisation, communication and behaviour problems, and conduct disorder is described with violating social norms with the use of aggression and misunderstanding. This could be the explanation for this comorbidity.

Disruptive mood dysregulation disorder (DMDD) is a childhood condition of extreme irritability, anger, and frequent, intense temper outbursts (1). This brief report study detected 15 (9.87%) patients with this disorder. In one study of 582 children with autism 45.2% of them very often had DMDD symptoms (38). These findings encourage not only future research of the presence of DMDD in ASD population but also encourage improvement in diagnostic instruments and screening for DMDD symptoms in everyday practise.

Anxiety disorders evaluated among 2,121 children and adolescents with ASD in a meta-analytic review revealed the prevalence as 39.6% and 34.8% in fixed and random effects models, respectively (18). Another meta-analysis found 20% of anxiety disorders comorbid to ASD (15). Anxiety is a common psychiatric comorbidity in children with recent-onset epilepsy with structural brain abnormalities (39). In this brief report we found 12 (7.89%) patients with anxiety disorder and ASD that is below percentage of findings in meta-analyses. One patient had detected cortical abnormality of a brain, but this patient also had epilepsy and ADHD. Patients with anxiety disorder reported in this report were also comorbid with other psychiatric disorders. And their possible risk factor correlation will be one of the future research steps in this ongoing study.

It is estimated that 40–80% of individuals with autism spectrum disorder have co-occurring sleep disturbances (40). Literature also accentuates that insomnia is 10 times more frequent in children with ASD than in those without (41). In this brief study report we noticed the presence of insomnia disorder in 12 (7, 89%) patients with ASD.

Despite scarce research in elimination disorder in children with ASD, study results imply a higher prevalence of incontinence in children with ASD compared to typically developing children (42). Overall rates for incontinence were reported in four studies, ranging from 9.3% to 57% (42). In our study we found five (3.25%) patients with ASD and elimination disorder.

Depression is not an uncommon diagnosis in youth, affecting about 12% of adolescents in the general population (9). Despite the limits to recognise depression in children and adolescents with ASD, because of the communication deficits a study of 101 children with ASD, aged 4 to 9 years, showed a prevalence of depression that ranged from 6% (IQ < 70) to 19% (IQ ≥ 70) (43). In our study we found depression diagnosed in three (1.97%) patients. They were all treated pharmacologically.

In this brief research report psychiatric disorders in ASD patients, such as schizophrenia spectrum and psychotic disorders, bipolar disorder, obsessive-compulsive disorder, suicide and suicide attempts and ideation, were not found. Possible reasons are already mentioned: limitations of this study, due to its small sample, observational character, and medical records only insight. Future follow-up is needed and in addition, collection of missing data and analyses of the risk factor correlation.

This brief research report aimed to present and describe a referral centre experience of psychiatric comorbidities in ASD population in one middle-income country. Autism Centre in Montenegro is a young medical institution that has ambition to give high-quality health service for their patients. It is of enormous importance to meliorate the diagnostic evaluation for all the possible symptomatology patterns in order to provide better treatment. This is the reason why investigating psychiatric comorbidities in children with ASD for us is crucial.

## Data Availability Statement

The raw data supporting the conclusions of this article will be made available by the authors, without undue reservation.

## Ethics Statement

The studies involving human participants were reviewed and approved by Ethical Committe of Clinical Centre of Montenegro. Written informed consent to participate in this study was provided by the participants' legal guardian/next of kin.

## Author Contributions

II created the study, collected and analysed the data, and wrote the manuscript.

## Conflict of Interest

The author declares that the research was conducted in the absence of any commercial or financial relationships that could be construed as a potential conflict of interest.

## References

[1] American Psychiatric Association. Diagnostic and Statistical Manual of Mental Disorders. 5th ed. American Psychiatric Association (2013).

[2] Elsabbagh M Divan G Koh YJ Kim YS Kauchali S Marcín C. Global prevalence of autism and other pervasive developmental disorders. Autism research. (2012) 5:160–79. 10.1002/aur.239 PMC3763210 22495912

[3] Croen LA Najjar DV Ray GT Lotspeich L Bernal P. A comparison of health care utilization and costs for children with and without autism spectrum disorder in a large group-model health plan. Pediatrics. (2006) 118:e1203–211. 10.1542/peds.2006-0127 17015508

[4] Lecavalier L. Behavioral and emotional problems in young people with pervasive developmental disorders: relative prevalence, effects of subject characteristics, empirical classification. J Autism Dev Disord. (2006) 36:1101–14. 10.1007/s10803-006-0147-5 16897387

[5] Maskey M Warnell F Parr JR Le Couteur A McConachie H. Emotional and behavioural problems in children with autism spectrum disorder. J Autism Dev Disord. (2013) 43:851–9. 10.1007/s10803-012-1622-9 22895777

[6] Brereton AV Tonge BJ Einfeld SL. Psychopathology in children and adolescents with autism compared to young people with intellectual disability. J. Autism Dev Disord. (2006) 36:863–70. 10.1007/s10803-006-0125-y 16897401

[7] Yerys BE Wallace GL Jankowski KF Bollich A Kenworthy L. Impaired consonant trigrams test (CTT) performance relates to everyday working memory difficulties in children with autism spectrum disorders. Child Neuropsychol. (2011) 17:391–9. 10.1080/09297049.2010.547462 21390918 PMC3424610

[8] Romero M Aguilar JM Del-Rey-Mejías Á Mayoral F Rapado M Peciña M. Psychiatric comorbidities in autism spectrum disorder: a comparative study between DSM-IV-TR and DSM-5 diagnosis. Int J Clin Heal Psychol. (2016) 16:266–75. 10.1016/j.ijchp.2016.03.001 30487870 PMC6225088

[9] DeFilippis M. Depression in children and adolescents with autism spectrum disorder. Children. (2018) 5:112. 10.3390/children5090112 30134542 PMC6162511

[10] Hossain MM Khan N Sultana A Ma P McKyer E Ahmed HU. Prevalence of comorbid psychiatric disorders among people with autism spectrum disorder: an umbrella review of systematic reviews and meta-analyses. Psychiatry Res. (2020) 287:112922. 10.1016/j.psychres.2020.112922 32203749

[11] Lugo-Marín J Magán-Maganto M Rivero-Santana A Cuellar-Pompa L Alviani M Jenaro-Rio C. Prevalence of psychiatric disorders in adults with autism spectrum disorder: a systematic review and meta-analysis. Res Autism Spectr Disord. (2019) 59:22–33. 10.1016/j.rasd.2018.12.004

[12] Richa S Fahed M Khoury E Mishara B. Suicide in autism spectrum disorders. Arch Suicide Res. (2014) 18:327–39. 10.1080/13811118.2013.824834 24713024

[13] De Giorgi R De Crescenzo F D'Alò GL Rizzo Pesci N Di Franco V. Prevalence of non-affective psychoses in individuals with autism spectrum disorders: a systematic review. J Clin Med. (2019) 8:1304. 10.3390/jcm8091304 31450601 PMC6780908

[14] Hedley D Uljarević M. Systematic review of suicide in autism spectrum disorder: current trends and implications. Curr Dev Disord Rep. (2018) 5:65–76. 10.1007/s40474-018-0133-6

[15] Lai M-C Kassee C Besney R Bonato S Hull L Mandy W Szatmari P. Prevalence of co-occurring mental health diagnoses in the autism population: a systematic review and meta-analysis. Lancet Psychiatry. (2019) 6:819–29. 10.2139/ssrn.3310628 31447415

[16] Zahid S Upthegrove R. Suicidality in autistic spectrum disorders: a systematic review. Cris J Cris Interv Suicide Prev. (2017) 38:237–46. 10.1027/0227-5910/a000458 28468556

[17] Hollocks MJ Lerh JW Magiati I Meiser-Stedman R Brugha TS. Anxiety and depression in adults with autism spectrum disorder: a systematic review and meta-analysis. Psychol Med. (2019) 49:559–72. 10.1017/S0033291718002283 30178724

[18] Van Steensel FJA Bögels SM Perrin S. Anxiety disorders in children and adolescents with Autistic spectrum disorders: a meta-analysis. Clin Child Fam Psychol Rev. (2011) 14:302–17. 10.1007/s10567-011-0097-0 21735077 PMC3162631

[19] Hannon G Taylor EP. Suicidal behaviour in adolescents and young adults with ASD: findings from a systematic review. Clin Psychol Rev. (2013) 33:1197–204. 10.1016/j.cpr.2013.10.003 24201088

[20] Segers M Rawana J. What do we know about suicidality in autism spectrum disorders? A systematic review. Autism Res. (2014) 7:507–21. 10.1002/aur.1375 24798640

[21] Padgett FE Miltsiou E Tiffin PA. The co-occurrence of nonaffective psychosis and the pervasive developmental disorders: a systematic review. J Intellect Dev Disabil. (2010) 35:187–98. 10.3109/13668250.2010.494596 20809880

[22] Skokauskas N Gallagher L. Psychosis, affective disorders and anxiety in autistic spectrum disorder: prevalence and nosological considerations. Psychopathology. (2009) 43:8–16. 10.1159/000255958 19893339

[23] Zheng Z Zheng P Zou X. Association between schizophrenia and autism spectrum disorder: a systematic review and meta-analysis. Autism Res. (2018) 11:1110–9. 10.1002/aur.1977 30284394

[24] Stewart ME Barnard L Pearson J Hasan R O'Brien G. Presentation of depression in autism and Asperger syndrome: a review. Autism. (2006) 10:103–16. 10.1177/1362361306062013 16522713

[25] van Steensel FJA Heeman EJ. Anxiety levels in children with autism spectrum disorder: a meta-analysis. J Child Fam Stud. (2017) 26:1753–67. 10.1007/s10826-017-0687-7 28680259 PMC5487760

[26] Hudson CC Hall Harkness L KL. Prevalence of depressive disorders in individuals with autism spectrum disorder: a meta-analysis. Abnorm Child Psychol. (2019) 47:165–75. 10.1007/s10802-018-0402-1 29497980

[27] Menezes M Robinson L Sanchez MJ Cook B. Depression in youth with autism spectrum disorders: a systematic review of studies published between 2012 and 2016. Rev J Autism Dev Disord. (2018) 5:370–89. 10.1007/s40489-018-0146-4

[28] Wigham S Barton S Parr JR Rodgers J. A systematic review of the rates of depression in children and adults with high-functioning autism spectrum disorder. J Ment Health Res Intellect Disabil. (2017) 10:267–87. 10.1080/19315864.2017.1299267

[29] Lecavalier L McCracken CE Aman MG McDougle CJ McCracken JT Tierney E. An exploration of concomitant psychiatric disorders in children with autism spectrum disorder. Compr Psychiatry. (2019) 88:57–64. 10.1016/j.comppsych.2018.10.012 30504071 PMC6295217

[30] Bishop DVM. Which neurodevelopmental disorders get researched and why? PLoS ONE. (2010) 5:e15112. 10.1371/journal.pone.0015112 21152085 PMC2994844

[31] Bitta M Kariuki SM Abubakar A Newton CRJC. Burden of neurodevelopmental disorders in low and middle-income countries: a systematic review and meta-analysis. Wellcome Open Res. (2018) 2:121. 10.12688/wellcomeopenres.13540.2 29881784 PMC5964629

[32] Baio J Wiggins L Christensen DL Maenner MJ Daniels J Warren Z. Prevalence of autism spectrum disorder among children aged 8 Years -Autism and developmental disabilities monitoring network, 11 Sites, United States, 2014. MMWR Surveill Summ. (2018) 67:1–23. 10.15585/mmwr.ss6706a1 29701730 PMC5919599

[33] Sayal K Prasad V Daley D Ford T Coghill D. ADHD in children and young people: prevalence, care pathways, andservice provision. Lancet Psychiatry. (2018) 5:175–86. 10.1016/S2215-0366(17)30167-0 29033005

[34] Davis NO Kollins SH. Treatment for co-occurring attention deficit/hyperactivity disorder and autism spectrum disorder. Neurotherapeutics. (2012) 9:518–30. 10.1007/s13311-012-0126-9 22678458 PMC3441928

[35] Gnanavel S Sharma P Kaushal P Hussain S. Attention deficit hyperactivity disorder and comorbidity: a review of literature. World J Clin Cases. (2019) 7:2420–6. 10.12998/wjcc.v7.i17.2420 31559278 PMC6745333

[36] Williams AE Giust JM Kronenberger WG Dunn DW. Epilepsy and attention-deficit hyperactivity disorder: links, risks, and challenges. Neuropsychiatr Dis Treat. (2016) 12:287–96. 10.2147/NDT.S81549 26929624 PMC4755462

[37] Bush G Valera EM Seidman LJ. Functional neuroimaging of attention-deficit/hyperactivity disorder: a review and suggested future directions. Biol Psychiatry. (2005) 57:1273–84. 10.1016/j.biopsych.2005.01.034 15949999

[38] Mayes SD Waxmonsky JD Calhoun SL Bixler EO. Disruptive mood dysregulation disorder symptoms and association with oppositional defiant and other disorders in a general population child sample. J Child Adolesc Psychopharmacol. (2016) 26:101–6. 10.1089/cap.2015.0074 26745442 PMC4800381

[39] Jones JE Jackson DC Chambers KL Hsu DA Stafstrom CE. Children with epilepsy and anxiety: subcortical and cortical differences. Epilepsia. (2015) 56:283–90. 10.1111/epi.12832 25580566 PMC4340751

[40] Sivertsen B Posserud MB Gillberg C Lundervold AJ Hysing M. Sleep problems in children with autism spectrum problems: a longitudinal population-based study. Autism. (2012) 16:139–50. 10.1177/1362361311404255 21478225

[41] Souders MC Mason TB Valladares O Bucan M Levy SE Mandell DS. Sleep behaviors and sleep quality in children with autism spectrum disorders. Sleep. (2009) 32:1566–78. 10.1093/sleep/32.12.1566 20041592 PMC2786040

[42] Niemczyk J Wagner C von Gontard A. Incontinence in autism spectrum disorder: a systematic review. Eur Child Adolescent Psychiatry. (2018) 27:1523–37. 10.1007/s00787-017-1062-3 29019014

[43] Salazar F Baird G Chandler S Tseng E O'sullivan T Howlin P. Co-occurring psychiatric disorders in preschool and elementary school-aged children with autism spectrum disorder. J Autism Dev Disord. (2015) 45:2283–22. 10.1007/s10803-015-2361-5 25737019

